# The Microbiome and Protein Carbamylation: Potential Targets for Protein-Restricted Diets Supplemented with Ketoanalogues in Predialysis Chronic Kidney Disease

**DOI:** 10.3390/nu15163503

**Published:** 2023-08-08

**Authors:** Valentin Faerber, Katharina S. Kuhn, Liliana Garneata, Kamyar Kalantar-Zadeh, Sahir Kalim, Dominic S. Raj, Martin Westphal

**Affiliations:** 1 Department of Medical Scientific Affairs, Pharma and Nutrition, Fresenius Kabi Deutschland GmbH, 61352 Bad Homburg, Germany; katharina.kuhn@fresenius-kabi.com (K.S.K.); martin.westphal@fresenius-kabi.com (M.W.); 2 “Dr. Carol Davila” Teaching Hospital of Nephrology, 4 Calea Grivitei, Sector 1, 010731 Bucharest, Romania; lilianagarna@yahoo.com; 3 Division of Nephrology Hypertension and Kidney Transplantation, Department of Medicine, University of California Irvine (UCI), Orange, CA 90286, USA; kkz@hs.uci.edu; 4 Division of Nephrology, Department of Medicine, Massachusetts General Hospital, Harvard Medical School, Boston, MA 02114, USA; skalim@mgh.harvard.edu; 5 Division of Kidney Diseases and Hypertension, George Washington University School of Medicine, Washington, DC 20037, USA; draj@mfa.gwu.edu

**Keywords:** chronic kidney disease, diet, protein restricted, microbiome, dysbiosis, uremic toxin, urea, carbamylation, posttranslational protein modification

## Abstract

In chronic kidney disease (CKD), metabolic derangements resulting from the interplay between decreasing renal excretory capacity and impaired gut function contribute to accelerating disease progression and enhancing the risk of complications. To protect residual kidney function and improve quality of life in conservatively managed predialysis CKD patients, current guidelines recommend protein-restricted diets supplemented with essential amino acids (EAAs) and their ketoanalogues (KAs). In clinical studies, such an approach improved nitrogen balance and other secondary metabolic disturbances, translating to clinical benefits, mainly the delayed initiation of dialysis. There is also increasing evidence that a protein-restricted diet supplemented with KAs slows down disease progression. In the present review article, recent insights into the role of KA/EAA-supplemented protein-restricted diets in delaying CKD progression are summarized, and possible mechanistic underpinnings, such as protein carbamylation and gut dysbiosis, are elucidated. Emerging evidence suggests that lowering urea levels may reduce protein carbamylation, which might contribute to decreased morbidity and mortality. Protein restriction, alone or in combination with KA/EAA supplementation, modulates gut dysbiosis and decreases the generation of gut-derived uremic toxins associated, e.g., with cardiovascular disease, inflammation, protein energy wasting, and disease progression. Future studies are warranted to assess the effects on the gut microbiome, the generation of uremic toxins, as well as markers of carbamylation.

## 1. Introduction

Chronic kidney disease (CKD) is a devastating condition characterized by progressive, irreversible loss of kidney function over time [1]. CKD affects approximately 9% of the population worldwide, accounting for 1.2 million deaths in 2017, thus representing a substantial global health burden [2]. Since 1990, the prevalence of CKD has increased by 29.3%. The burden of CKD is disproportionately higher among the developing countries and among individuals in the lower socio-demographic Index [2]. It is expected that CKD will represent the 5th major cause of death worldwide by 2040 [3].

There are five progressive stages of CKD, which are assigned based on the decrease in patient’s glomerular filtration rate (GFR) and levels of albuminuria [1]. The signs and symptoms of CKD, affecting virtually all body systems and organs, are most often attributed to the accumulation of urea and uremic toxins, partially derived from protein and amino acid metabolism [4,5]. With progressive decline in GFR and associated accumulation of retention solutes, quality of life deteriorates and healthcare costs rise [6,7]. Ultimately, patients proceed to irreversible kidney failure, also referred to as end-stage kidney disease (ESKD), a condition requiring renal replacement therapy (RRT), either as maintenance dialysis or kidney transplantation to preserve life [8]. Dialysis treatment in turn is associated with a high risk of relevant complications, including cardiovascular disease (CVD), anemia, mineral bone disorder (MBD), chronic metabolic acidosis, and protein energy wasting (PEW) [1,5,9]. In light of the associated high morbidity and mortality, a major goal of CKD treatment is to slow its progression and delay the onset of dialysis [8,10].

If detected early, the progression of CKD to ESKD can be delayed or prevented through appropriate interventions [1]. To protect residual renal function and improve quality of life in predialysis CKD patients (stages 3–5), experts in the field recommend a dietary protein restriction to reduce uremia and the formation of uremic toxins, and to slow CKD progression, along with lowering the cardiovascular risk [8]. According to the National Kidney Foundation’s Kidney Disease Outcomes Quality Initiative (KDOQI) guidelines, either a low-protein diet (LPD) providing 0.55–0.60 g dietary protein/kg body weight/day, or a supplemented very low protein diet (sVLPD) providing 0.28–0.43 g dietary protein/kg body weight/day with the addition of a mixture of essential amino acids (EAAs) and ketoanalogues (KAs) to meet protein requirements (0.55–0.60 g/kg body weight/day) is recommended [11].

KAs serve as the precursors of the corresponding EAAs via conversion by transamination, i.e., the transfer of an amino group. This process uses amino groups from circulating AA, thus preventing their incorporation into urea or other potentially toxic nitrogenous waste products. KA can thus contribute to maintaining an adequate supply of EAAs for protein synthesis and other metabolic pathways and reduce nitrogen load without an associated adverse effect on azotemia [12].

The present review article summarizes recent insights into the role of protein-restricted diets supplemented with KAs in delaying CKD progression in predialysis CKD patients as well as elucidates possible mechanistic underpinnings.

## 2. Protein-Restricted Diets with KAs/EAAs: Effects on CKD Progression

Garneata et al. (2016) [13] conducted a randomized controlled trial (RCT) with non-diabetic adults suffering from progressed CKD (stages 4–5; eGFR < 30 mL/min) to compare a vegetarian KA/EAA-supplemented sVLPD with an LPD on the progression of CKD and requirement for RRT (composite endpoint of need for RRT or halving of the initial eGFR at any timepoint during the study). The results showed that patients on sVLPD had higher adjusted event-free survival rates, a slower decline in estimated GFR (eGFR), and less need for RRT compared to those on LPD. Moreover, there was no difference in any of the parameters of nutritional status versus baseline or versus LPD, and no adverse reactions to VLPD or Kas were noted. Importantly, the achieved protein intake was closely monitored and remained very close to prescription throughout the study and remained stable throughout the study (median 0.29 and 0.59 g/kg per day, respectively, at the end of the study). Only 3% of patients dropped out of the study prematurely, without any difference between groups. The authors thus concluded that a vegetarian VLPD supplemented with KAs was nutritionally safe and delayed dialysis initiation in patients with eGFR < 20 mL/min by ameliorating CKD-associated metabolic disturbances [13]. Long-term follow-up of these patients (median time of follow-up was 10.5 years) showed that patient survival was higher among those following sVLPD compared to the LPD (96% vs. 82%). Only the type of nutritional intervention was associated with the survival advantage. Moreover, significantly less patients in the sVLPD group required KRT at follow-up (51% versus 93%). In patients (still) not on RRT, the adherence to the nutritional intervention remained very good throughout the follow-up in both groups, and there were no changes in the nutritional status in any arm [14]. A recent meta-analysis confirmed that interventions combining protein restriction with KA/EAA supplementation had a significant role in delaying CKD progression [15].

Bellizzi et al. [16] reported results from the pragmatic, randomized ERIKA study that aimed to compare effects of an sVLPD supplemented with EAAs and KAs versus a LPD on outcome in patients with chronic kidney disease (CKD) and concluded that neither the primary outcome, time to renal death, nor mortality risk differed between treatment groups. However, the poor adherence to the prescribed protein restriction should be noted since the achieved protein intake was higher than the prescribed one throughout the study, raising concerns about the dietary monitoring. It is also noteworthy that approximately 25% of patients in the ERIKA study were moderately or severely malnourished. It has been suggested that severely malnourished patients should not receive an sVLDP due to the probable lack of appetite and consequent risk of exacerbating malnutrition [17]. Clinically stable patients with CKD stages 3–5 can efficiently adapt their muscle protein turnover to an LPD containing 0.55–0.6 g protein/kg or a supplemented very low protein diet (VLPD) by decreasing muscle protein degradation and increasing the efficiency of muscle protein turnover [18].

Overall, the majority of the available evidence underpins that protein-restricted diets supplemented with KAs are an effective strategy to delay CKD progression while maintaining nutritional status in predialysis patients (Table 1). In the following sections, we will shed more light on the underlying mechanisms of action.

## 3. Impact of Urea on CKD

As CKD progresses, increasing retention and accumulation of urea is observed due to the decreasing ability of the kidneys to excrete metabolites from protein breakdown. Increasing evidence suggests a range of direct toxic effects of urea which have been linked to cardiovascular damage. These include, but are not restricted to, the induction of molecular changes related to insulin resistance, increased production of radical oxygen species (ROS) and other inflammatory mediators, the induction of apoptosis, the disruption of intestinal barrier function, and the increased generation of carbamylated compounds [19,20,21]. In a large prospective cohort study in predialysis CKD patients, higher serum urea levels were found to be associated with a higher risk of fatal and non-fatal cardiovascular events as well as with a higher risk of death before RRT initiation, indicating that urea could be a key factor predicting cardiovascular risk in patients with CKD [22].

**Figure 1:**
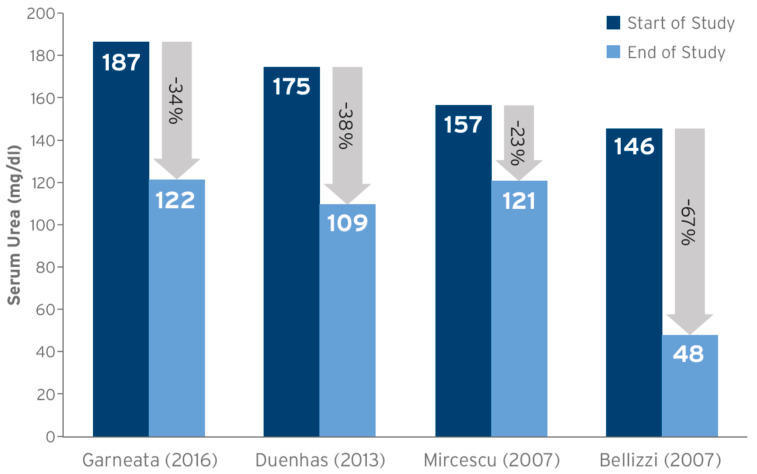
Summary of studies examining the effect of a KA/EAA-supplemented VLPD on serum urea levels from study start to study end. Sources: Mircescu (2007) [24]; Duenhas (2013) [23]; Garneata (2016) [13]; Bellizzi (2007) [25].

Studies showing benefits of KA/EAA-supplemented protein-restricted diets in predialysis CKD patients have also shown considerable reductions in serum urea levels [13,23,24,25,26,27] (Figure 1). In the study by Bellizzi et al. (2018) [28] that examined the metabolic effects of an sLPD among CKD patients (non-dialysis, stages 3–5, N = 197), serum urea significantly decreased after 6 months both among patients with (from 131 ± 58 to 105 ± 49 mg/dL, *p* < 0.05) and without diabetes (from 115 ± 52 to 88 ± 36 mg/dL, *p* < 0.05). In a meta-analysis by Rhee et al. (2018) [29], 1-year serum urea values trended lower in those patients who received Ketosteril-sVLPD vs. LPD (three studies, weighted mean difference (WMD) −55.30, 95% confidence interval (CI) −117.54 to 6.95).

**Figure 2:**
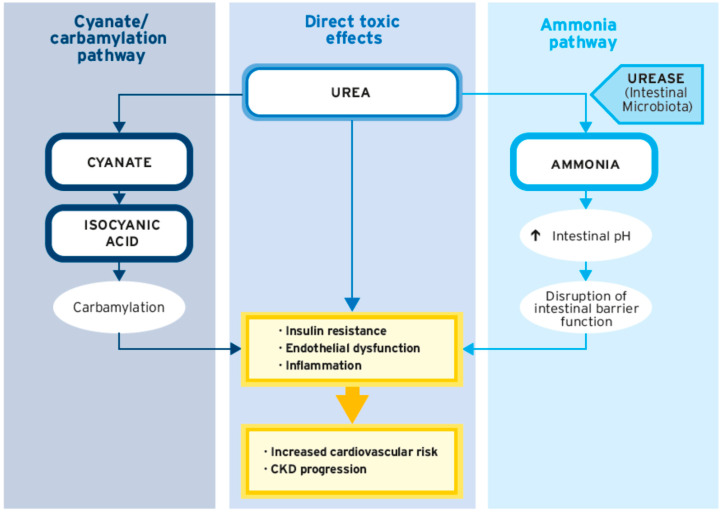
Pathways involved in the toxicity of urea.

Apart from the direct toxic effects of urea, its degradation products cyanate and ammonia can interfere with biochemical and organ functions (Figure 2). Due to the accumulation of urea, levels of its dissociation product cyanate are elevated in CKD [19,30]. High levels of cyanate increase CVD risk by inducing endothelial dysfunction in CKD patients [31]. Moreover, cyanate induces the generation of carbamylated compounds that interfere with organ and body function [19]. As reported by di Iorio et al., a considerable reduction in urea levels (up to 61%) with the use of an sVLPD was accompanied by a reduction in cyanates of about 20–30% [27]. Accordingly, it is conceivable that the observed benefits of s(V)LPDs in CKD patients are closely linked to the associated decrease in urea, thereby reducing cyanate generation and protein carbamylation.

## 4. Impact of Protein Carbamylation on CKD Progression

### 4.1. Carbamylation—Definitions and Pathophysiological Mechanisms

Carbamylation refers to the nonenzymatic posttranslational modification of proteins in the blood through the transfer of a carbamoyl group from cyanate, driven by a variety of factors, e.g., inflammation, kidney disease, diet, smoking, and air pollution [27,32,33]. In CKD, carbamylation is mainly due to the exposure to cyanate derived from the dissociation of urea [32]. Under physiological conditions, a small amount of urea (<1%) spontaneously dissociates into ammonium ions and cyanate. As kidney function declines, urea accumulates in the blood, so the burden of carbamylation rises [19,33].

Carbamylation affects the functionality of numerous organs and tissues in the human body and has been associated with memory deficits, aging, impaired vision, atherosclerosis, congestive heart failure, disturbed hematopoiesis and coagulation, autoimmune disease, and kidney fibrosis [32]. Carbamylated proteins interfere with organ and body functions through multiple mechanisms [19,32]. Carbamylated lipoproteins, collagen, fibrin, proteoglycans, and fibronectin contribute to atherosclerosis and cardiovascular risk [32,33,34]. Carbamylation of collagen or enzymes involved in extracellular matrix remodeling may accelerate the progression of CKD by enhancing urea-associated fibrosis, leading to a detrimental positive feedback loop [32]. Carbamylation of erythropoietin deactivates its erythropoietic activity, and therefore, carbamylation contributes to the pathophysiology of anemia in CKD [32,35]. Moreover, carbamylation has been proposed to be involved in the pathogenesis of CKD, CVD, and other chronic diseases such as rheumatoid arthritis via the induction of an autoantibody response through the formation of anti-carbamylated protein (anti-CarP) antibodies [36,37].

### 4.2. Carbamylation Is Associated with CKD Progression and Mortality in CKD Patients

Patients in the advanced stages of CKD have a high risk of major cardiovascular events, and the excess cardiovascular mortality cannot entirely be explained by traditional risk factors [38]. Besides oxidative stress and systemic inflammation, mechanisms to explain the excess CVD burden associated with CKD include uremic toxins and increased carbamylation [38]. It is currently assumed that protein carbamylation and cyanate compounds represent an important link between CKD and CVD [27]. Several studies revealing a link between carbamylation and cardiovascular mortality are summarized in Table 2. While the majority of epidemiological carbamylation studies have focused on ESKD patients [39,40,41,42], Kalim et al. investigated the association between carbamylation load and CKD progression and mortality in pre-dialysis CKD patients. In two studies, levels of carbamylated albumin were found to be predictive of CKD progression and mortality independent of established predictors such as eGFR and proteinuria [43,44].

### 4.3. Protein-Restricted Diets with KAs/EAAs Reduce Carbamylation in CKD Patients

Di Iorio et al. (2018) [27] conducted a crossover RCT in adult patients with moderate CKD (stages 3B–4) to verify the hypothesis that carbamylation is enhanced in patients with higher urea levels and that protein-restricted diets, either a Mediterranean diet (MD) or KA/EAA-supplemented sVLPD, are able to reduce carbamylation (Table 3). Compared to a free diet (FD), both dietary interventions significantly reduced the serum level of homocitrulline, a marker of overall carbamylation. Importantly, homocitrulline and protein carbamylation levels were significantly correlated with serum urea levels (R2 = 0.5; *p* < 0.0001). The authors concluded that decreasing urea levels, e.g., with an sVLPD, can reduce protein carbamylation as well as cyanate production from urea. Notably, however, bolstering serum free amino acids also appears to reduce carbamylation, as was demonstrated in hemodialysis patients receiving intradialytic amino acid therapy on dialysis [45]. In this study, over an 8-week period, nutritionally intact hemodialysis patients receiving intradialytic amino acids showed a significant reduction in their carbamylated albumin levels when compared to controls undergoing routine care [45].

## 5. Role of the Gut Microbiome in CKD Patients

Nephrologists have explored the intestine as a “substitute kidney”. It is well known that gut assumes an increasing role in nitrogen waste excretion to compensate for the loss of kidney function. Now, it is becoming evident that gut microbiota contributes to protein and energy metabolism. The degradation of urea by urease-expressing colonic bacteria gives rise to increased ammonia concentrations in the gut, contributing to intestinal barrier dysfunction and increased inflammation (Figure 2) [19,48]. Moreover, the implications of a dysregulated gut microbiome in the pathophysiology and progression of CKD, an interrelation that has been designated as the “gut-kidney axis”, are increasingly recognized [49,50].

### 5.1. The Gut Microbiome in CKD—Why We Should Care

The human gut comprises approximately 1014 microorganisms that play a pivotal role in human health and disease [50,51,52,53,54]. These commensal microorganisms perform several physiological functions such as modulating immunity, protecting against pathobionts, regulating endogenous metabolism of carbohydrates, lipids, and proteins, and biosynthesis of vitamins and amino acids, thus contributing to nutritional balance [50,55]. Containing at least 100 times more genes than the human genome, the gut microbiome has been designated as the “second genome” [53,56,57]. Abundance and diversity of bacteria increase from the proximal to the distal regions of the intestine. While the proximal colon is predominantly colonized by saccharolytic bacteria, proteolytic bacteria are most abundant in the distal part [58]. Since proteolysis can result in the generation of toxic compounds, this distribution has physiological significance in minimizing the intestinal contact time with these substances, thereby reducing the capacity for absorption. Emerging data also indicate that the microbiota may also be involved in the post-translational modification of proteins and amino acid [59,60].

### 5.2. The Interrelation between Gut Dysbiosis and CKD

Gut dysbiosis has been broadly defined as an “imbalance in the intestinal microbial community with quantitative and qualitative changes in the composition and metabolic activities of the gut microbiota” [54]. Dysregulation of the gut microbiota in CKD patients appears to promote CKD progression through alterations in immune response, blood pressure regulation, and metabolic changes [61,62,63,64]. In this context, Gao et al. found that, with increasing CKD stage, butyrate producers decreased, whereas Methanobacteria and several Collinsella species associated with atherosclerosis risk increased. In addition, an inverse association with kidney function was found for microbial genera linked to the production of SCFAs and bile acid deconjugation. These factors, in turn, have been inversely related to CVD risk [63].

The main contributing factors to gut dysbiosis in patients with CKD include slow intestinal transit time, impaired protein digestion and absorption, decreased consumption of dietary fiber, iron therapy, and frequent use of antibiotics [53]. When reviewing the above studies together, it appears reasonable that initial adaptive changes in gut microbiota become maladaptive in later stages of CKD, exacerbating CKD-related complications [54].

### 5.3. Gut Dysbiosis Is Associated with Increased Production of Uremic Toxins

Diet remains the single most important modulator of gut microbiome in health, which adaptively changes their community structure and function [62,65]. In uremia, due to impaired protein digestion and absorption, increased amounts of undigested protein reach the distal part of the colon, favoring the proliferation of proteolytic bacteria [53]. Enhanced proteolysis in the colon significantly contributes to the generation of uremic toxins [49,50,66,67]. Several well-described uremic toxins are derived from the metabolism of phenolic compounds or aromatic AAs by gut bacteria, including indoles such as indoxyl sulfate (IS) and indole acetic acid (IAA), phenols such as para-cresol sulfate (PCS), phenylacetylglutamine (PAG) and hippurate, and polyamines such as putrescine, agmatine, cadaverine, tyramine, and histamine [53]. Moreover, metabolism of dietary phosphatidylcholine results in the formation of choline, betaine, and trimethylamine (TMA), a precursor for the hepatic synthesis of trimethylamine-N oxide (TMAO) [68]. Impaired intestinal barrier function in CKD permits the translocation of gut-derived uremic toxins into the systemic circulation [53]. While under normal conditions uremic toxins are largely excreted by healthy kidneys, they are retained when renal clearance decreases in CKD [20,69].

### 5.4. Uremic Toxins Are Associated with Disease Progression and Cardiovascular Risk in CKD

Clinical manifestations of increased levels of gut-derived uremic toxins include CVD, inflammation, fibrosis, endocrine, metabolic and neurologic disorders, protein energy wasting (PEW), and the progression of CKD [20,53]. An increasing body of evidence suggests that uremic toxins generated by a dysbiotic gut microbiome contribute to the progression to CKD and associated cardiovascular complications [67,70]. In nondialysis CKD patients, serum levels of IS and PCS were shown to be predictive of CKD progression, and PCS was also associated with all-cause mortality [71]. IS and PCS are considered critical factors in the pathophysiology of CKD-associated immune dysfunction, accounting for the increased risk of bacterial infections and damage to endothelial cells [72]. Among older patients with advanced CKD, IS and PCS were shown to be associated with inflammatory cytokines that may influence nutritional status, and PCS was positively associated with PEW [73]. TMAO has been linked to end-organ dysfunction, increased CVD risk, and mortality in CKD [67,68,74,75]. Moreover, several investigators have found associations between increased CVD risk and levels of TMAO [68,74].

### 5.5. Dietary Interventions with Protein Restricetd Diets and/or KA/EAA Supplementation: Effects on Gut Microbiota and Generation of Uremic Toxins

Protein restriction is currently discussed as a suitable therapeutic approach to beneficially modulate the gut microbiota in CKD patients [76]. The quantity and quality of dietary protein strongly influence the microbial diversity and abundance of selected bacterial genera and species in the gut [52]. Notably, a high dietary protein intake has been linked to the dysregulation of saccharolytic bacteria and increased abundance of proteolytic bacteria, resulting in an increased microbial production of proteolysis-derived uremic toxins such as PCS, IS, and TMAO [62,77]. Moreover, the source of protein intake should also be considered. A diet high in animal protein, especially red meat and eggs, can contribute to an increase in TMAO production, as it provides high amounts of the TMAO precursors choline and L-carnitine [77].

Mice fed with a diet low in protein content, but rich in fiber, had a preponderance of short-chain fatty acid (SCFA)-producing saccharolytic bacteria. SCFA are involved in energy homeostasis, maintaining gut barrier, blood pressure control, and immune regulation. Furthermore, mice consuming a high-protein/low-fiber diet were enriched for proteolytic bacteria and exhibited a decrease in Th17 polarization and an increase in Treg cell commitment compared to mice fed with an HP-LF diet [78].

**Figure 3:**
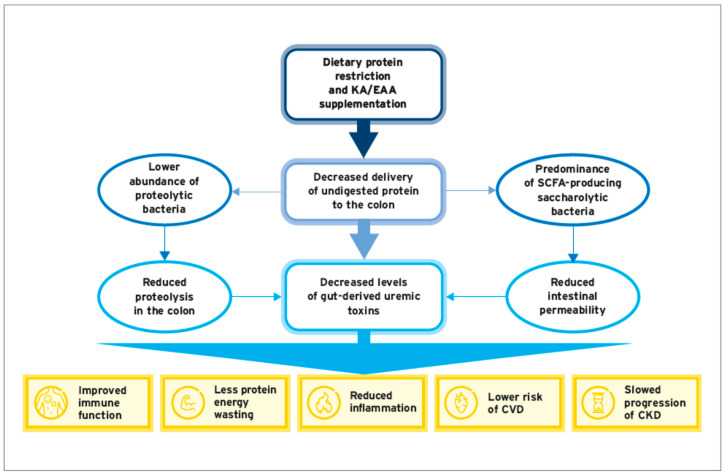
Protein restriction and KA/EAA supplementation: effects on gut microbiota, generation of uremic toxins, and associated clinical manifestations.

A longitudinal study in stage 3–4 CKD patients evaluated the effects of an LPD on the serum levels of uremic toxins and the gut microbiota profile [79]. The study showed a significant decrease in serum levels of PCS after 6 months in patients, with good adherence to the LPD as compared to nonadherent patients. A change in the gut microbiota profile was observed after nutritional intervention in both groups. The average number of bands was positively associated with protein intake, suggesting that the amount of protein present in the diet modulates the composition of the gut microbiota. In addition, total and LDL cholesterol levels were reduced in adherent patients, while there was no deleterious effect on nutritional status due to protein restriction [79]. It is thus conceivable that protein restriction represents a viable strategy to reduce the production of uremic toxins by the gut microbiota in predialysis CKD patients (Figure 3).

In a rat model of CKD, treatment with KAs/EAAs (1.6 g/kg/day by intragastric administration) resulted in a beneficial modification of the gut microbiota associated with less intestinal barrier injury and decreased serum concentrations of IS, betaine, choline, and cholesterol. Moreover, KA/EAA treatment reduced serum creatinine and blood urea nitrogen (BUN), reduced proteinuria, and alleviated histological damage to the kidneys [80]. These findings suggest that KAs may exert marked effects on gut microbiota and barrier function in CKD, which could translate into improved serum metabolic profiles and reduced kidney injury [80].

The effects of a KA/EAA-supplemented sVLPD compared to a Mediterranean diet (MD) on the modulation of gut microbiota, intestinal permeability, and levels of uremic toxins were investigated by di Iorio et al. (2019) in a cross-over RCT in adult patients with moderate CKD (stages 3B–4) (Table 3) [46]. With the sVLPD, a beneficial modulation of the gut microbiota was seen. The abundance of Proteobacteria associated with inflammation was reduced, whereas butyrate-producing species were increased. Reduced levels of serum lactate compared to FD and MD, respectively, indicated a reduction in intestinal permeability, and lactate levels were positively correlated with BUN [46]. Moreover, with the sVLPD, levels of total and free IS and PCS were significantly reduced after 6 months, both compared to the MD and the FD [46].

Two years later, Rochetti et al. [47] investigated the specific effect of the added KAs by adding a further treatment group receiving a KA/EAA-supplemented MD to the above study design. Compared to an unsupplemented MD, KA/EAA supplementation resulted in a profound modulation of the intestinal microbiota (Table 1). Levels of total and free IS and PCS were significantly lower after the supplemented MD than after the FD. The reduction in uremic toxins was also greater, but not significant, compared to unsupplemented MD, and smaller compared to sVLPD. Intestinal permeability was not reduced any further with the supplemented compared to the unsupplemented MD, and it was positively correlated with BUN levels. These findings underpin the driving role of urea reduction in restoring gut integrity. Overall, the findings indicate that KAs/EAAs and protein restriction act synergistically in the modulation of gut microbiota.

## 6. Conclusions

The gut microbiome plays a key role in many metabolic processes that have a decisive influence on the course of CKD progression. Since gut dysbiosis contributes to the progression of CKD and the occurrence of cardiovascular complications, the microbiome has emerged as a promising therapeutic target in predialysis CKD patients. Emerging evidence indicates that protein-restricted diets supplemented with combinations of KAs and EAAs are effective in modulating the gut microbiota, restoring intestinal integrity, and reducing the production of uremic toxins (Table 3). Apparently, these effects are mediated via a reduction in urea load, mainly due to protein restriction.

A further mechanistic underpinning related to CKD progression and its associated complications, e.g., anemia, and increased cardiovascular risk, is increased protein carbamylation. There is evidence from an RCT [27] that lowering azotemia by means of an sVLPD can reduce cyanate production and protein carbamylation in predialysis CKD patients (Table 3). This suggests that indicators of protein carbamylation could serve as a sensitive biomarker to assess adherence to and benefits of protein-restricted diets supplemented with KAs/EAAs.

Future studies are warranted to investigate the role of protein-restricted diets supplemented with KAs/EAAS on the gut microbiome, generation of uremic toxins, and markers of carbamylation in predialytic CKD patients.

## Figures and Tables

**Table 1:** Studies investigating effects of KA/EAA-supplemented protein-restricted diets on disease progression in patients with predialysis CKD.

Study	Design	Population	Intervention	Main Outcomes
Garneata, 2016 [13]	RCT, open-label	CKD stages 3–4, non-diabetic	sVLPD (0.3 g/kg IBW/day) + KA/EAA (0.125 g/kg IBW/day) vs. LPD (0.6 g/kg IBW/day) 15 months	sVLPD vs. LPD: Higher adjusted event-free survival rates; Fewer patients required KRT; eGFR declined less; Urea and uric acid serum levels were lowered; No difference in markers of nutritional status. Very good compliance
Garneata, 2019 [14]	RCT, open-label, long-term follow-up	CKD stages 3–4, non-diabetic	sVLPD (0.3 g/kg IBW/day) + KA/EAA (0.125 g/kg IBW/day) vs. LPD (0.6 g/kg IBW/day) 5 years	sVLPD vs. LPD: Improved 5-year survival (96% vs. 82%); Fewer patients required KRT at follow-up; No difference in markers of nutritional status. Very good compliance Only the type of nutritional intervention was associated with the survival advantage
Bellizzi et al., 2021 [16]	RCT, multicentre	CKD stages 4–5	sVLPD (prescribed 0.3 g protein/kg IBW/day) + KA/EAA (0.125 g/kg IBW/day) vs. LPD (prescribed: 0.6 g protein/kg IBW/day)	sVLPD vs. LPD: Similar time to renal death (primary outcome); No significant difference in risk of renal death, risks of ESKD or time to fatal/nonfatal cardiovascular events; Similar eGFR decline from baseline to 36 months; No changes in nutritional status in any group; Low patient compliance to diet: actual median protein intake was 0.60 g/kg IBW/d vs. 0.83 g/kg IBW/d.

RCT: randomized controlled trial, CKD: chronic kidney disease, LPD: low-protein diet, FD: free diet, MD: Mediterranean diet, s: supplemented, (e)GFR: (estimated) glomerular filtration rate, BW: body weight, IBW: ideal body weight, KA: ketoanalogue, EAA: essential amino acid, IS: indoxyl sulfate, PS: para-cresol sulfate, ESKD: end-stage kidney disease.

**Table 2:** Studies linking carbamylation to mortality.

Study	Design	Carbamylated Compound	Population	Associated Outcomes
Wang, 2007 [34]	Case–control	Protein-bound homocitrulline	Subjects undergoing cardiac catheterization	Risk of coronary artery disease, future myocardial infarction, stroke, and death
Koeth, 2013 [39]	Observational cohort	Protein-bound homocitrulline	ESKD patients on hemodialysis	5-year risk of death
Berg, 2013 [42]	Observational cohort	Carbamylated albumin	ESKD patients on hemodialysis with and without diabetes	1-year risk of death
Drechsler, 2015 [41]	Observational cohort	Carbamylated albumin	ESKD patients on hemodialysis with diabetes	1-year adjusted risk of overall and cardiovascular mortality, and sudden cardiac death at 4 years, with additional risk of death from congestive heart failure
Kalim, 2016 [40]	Observational cohort	Carbamylated albumin	ESKD patients on hemodialysis	1-year risk of death
Kalim, 2021 [43]	Two nested case–control studies	Carbamylated albumin	Nondialysis CKD patients	CKD progression 1-year risk of death (trend)

CKD: chronic kidney disease, ESKD: end-stage kidney disease.

**Table 3:** Studies investigating effects of KA/EAA-supplemented protein-restricted diets on uremic toxins, gut microbiota, and/or carbamylation in patients with predialysis CKD.

Study	Design	Population	Intervention	Main Outcomes
Di Iorio, 2018 [27]	RCT, crossover	CKD stages 3B–4	sVLPD (0.3 g protein/kg BW/day) vs. Mediterranean diet (MD, 0.7–0.8 g protein/kg BW/d Vs. Free diet (FD, 1 g protein/kg BW/day) 6 months	sVLPD vs. MD and FD - Lowered serum urea levels; - Lowered homocitrulline levels and homocitrulline/lysine ratios Homocitrulline and protein carbamylation levels were significantly correlated with serum urea levels.
Di Iorio 2019 [46]	RCT, crossover	CKD stages 3B–4	sVLPD (0.3 g protein/kg BW/day) vs. Mediterranean diet (MD, 0.7–0.8 g protein/kg BW/d Vs. Free diet (FD, 1 g protein/kg BW/day) 6 months	With the sVLPD vs. MD and FD: - Reduced Proteobacteria and increased Actinobacteria; - Reduction in intestinal permeability (associated with the decrease in BUN). With MD and sVLPD vs. FD: - Increase in Lachnospiraceae, Ruminococcaceae, Prevotellaceae, and Bifidobacteriaceae; - Reduced levels of total and free IS and PCS.
Rocchetti 2021 [47]	RCT, crossover	CKD stages 3B–4	sVLPD (0.3 g protein/kg BW/day) vs. Mediterranean diet (MD, 0.7–0.8 g protein/kg BW/d vs. Supplemented Mediterranean diet (sMD, 0.7–0.8 g protein/kg BW/d Vs. Free diet (FD, 1 g protein/kg BW/day) 6 months	With the sMD vs. MD: - Decrease in Clostridiaceae, Methanobacteriaceae, Prevotellaceae and Lactobacillaceae, and an increase in Bacteroidaceae and Lachnospiraceae; Levels of total and free IS and PCS were significantly lower after sMD than after FD. The reduction in uremic toxins with sMD was greater, but not significant, vs. MD, and smaller compared to sVLPD. Intestinal permeability was not reduced any further with the supplemented compared to the unsupplemented MD.

RCT: randomized controlled trial, CKD: chronic kidney disease, LPD: low-protein diet, FD: free diet, MD: Mediterranean diet, s: supplemented, (e)GFR: (estimated) glomerular filtration rate, BW: body weight, IBW: ideal body weight, KA: ketoanalogue, EAA: essential amino acid, IS: indoxyl sulfate, PS: para-cresol sulfate, ESKD: end-stage kidney disease.

## Data Availability

Not applicable.
